# Making multi-omics data accessible to researchers

**DOI:** 10.1038/s41597-019-0258-4

**Published:** 2019-10-31

**Authors:** Ana Conesa, Stephan Beck

**Affiliations:** 1 Microbiology and Cell Science Department, Institute for Food and Agricultural Research, Genetics Institute, University of Florida, Gainesville, Florida USA; 2 UCL Cancer institute, University College London, London, UK

**Keywords:** Genome, Data integration

## Abstract

A special collection on multi-omics data sharing, launched today at *Scientific Data*, offers to the scientific community a compendium of multi-omics datasets ready for reuse, which showcase the diversity of multi-omics projects and highlights innovative approaches for preprocessing, quality control, hosting and access.

Omics technologies are defined as high-throughput biochemical assays that measure comprehensively and simultaneously molecules of the same type from a biological sample. For example, genomics profile DNA, transcriptomics measure transcripts; proteomics and metabolomics quantify proteins and metabolites, respectively. The “omics” notion refers to the fact that all or nearly all instances of the targeted molecular space are measured in the assay, and therefore they provide holistic views of the biological system. Initially, omics experiments used to concentrate on one type of assay (i.e. transcriptomics) and provide *single-omics* data. However, more recently researchers have combined multiple assays from the same set of samples to create *multi-omics datasets*. The limited insights of early single omics projects, such as the Human Genome Project, and the proliferation of facilities that offer affordable omics assays on a service basis, have driven the development of many new multi-omics projects. The added power of multi-omics has been evident for some time, but the complexity of managing and integrating such multi-dimensional data continues to be a challenge. Data storage, quality control and statistical analysis are all more complex for these datasets, and compliance with the FAIR principles^1^ is inherently more difficult. Moreover, *complete multi-omics datasets* (where the same set of omics assays are obtained for all study samples) are difficult to generate. Consequently, researchers who may want to benefit from the multi-platform nature of these data frequently face limitations in accessing full data records or identifying suitable multi-omics datasets for their research questions.

In this Comment, we describe *Scientific Data’s* collection on multi-omic data and discuss how the authors of the included Data Descriptors have addressed these challenges for their datasets. Beyond the value of these datasets for deriving new biological and biomedical insights, this collection provides a novel opportunity for bioinformaticians and statisticians to access well-documented multi-omics datasets for the development of integrative analysis approaches.

## Description of the Collection

At time of launching this collection, six papers are included, representing a wide variety of experimental settings and scientific goals (Table 1). Three datasets deal with human diseases, eitherusing human samples (ColPortal^2^) or mouse models (Sleep Deprivation^3^, Fibrotic Kidney^4^), while another manuscript describes the pilot data of the UK Personal Genome Project (PGP-UK^5^). One dataset targets a plant model of virus infection (PVY^6^) and another paper models B-cell differentiation in mouse (STATegra^7^). Additionally, experimental designs include time series data for the multidimensional modeling of biological processes. Two papers profile human cohorts with multi-omics data: the PGP-UK manuscript does this on healthy individuals to describe human heterogeneity while ColPortal analyzes colorectal cancer samples to identify markers of disease subtypes. Though the combination of omics technologies is very variable, all Data Descriptors share the inclusion of gene expression data. Gene expression is further combined with genomics and epigenomics data (PGP-UK), epigenomics and microbiome (ColPortal), metabolomics (Sleep Deprivation), proteomics and microRNAs (PVY and Fibrotic Kindey) and nearly all of the above in the case of STATegra. In all studies, except for STATegra and Fibrotic Kidney, additional multivariate phenotypic data has been collected and presented together with the omics datasets.

**Table 1:** Overview of Data Descriptors in the *Scientific Data* multi-omics data collection.

Dataset	Omics	Experimenal Design	Organism	Sample	Block factor	Phentotypic data	Full multiomics data?	Raw data	Processed data	Scripts available	Name and Goal of specific software development
Stategra	RNA-seq, microRNA-seq, ChIP-seq, RRBS, Dnase-seq, ATAC-seq, scRNA-seq, scATAC-seq, proteomics, metabolomics	Time course, 3 replicates, 6 points, Treatment and Control Samples, 1 cell line	Mouse	B3 cell line	Cell culture. Different batches for different omics	NO	NO	GEO, Proteome-Xchange, Metabolomics,	STATegraKB, Lifebit, Figshare	YES	STATegraKB, Integrative analysis
Personal Genome Project-UK (PGP-UK)	RNA-seq, WGBS, 450 K Methylomics, WGS	10 individuals	Human	Blood	Individuals	YES	YES	ENA, ArrayExpress	Lifebit	YES	PGP-UK portal
ColPortal	Mehylation 450k arrays, 16 S sequencing, expression arrays, microRNA arrays	167 individuals, 3 histological cancer subtypes (Serrated, Convencional, hMSI), CR polyps and surrounding healthy tissue	Human	Colon tissue	Individuals	YES	NO	ENA, GEO	Figshare, ColPortal	YES	ColPortal, Integrative analysis
Potato Virus Y (PVY)	Expression arrays, smallRNA-seq, Degradome-seq, Proteomics	Time course, 7 time points, triplicates, 2 leaf types, 2 genotypes	Potato	Leaf	Plant	YES	NO	GEO	fairdomhub	YES	NA
Sleep Deprivation	RNA-seq, Metabolomics	Treatment-control; 32 genotypes, 3 bio replicates	Mouse	Cortex and Liver, (RNAs-seq). Plasma (metabolomics)	Individual	YES an uses HPO	YES	GEO, Figshare	Group’s server	YES	DGO, Analysis reproducibility
Fibrotic Kidney	RNA-seq, microRNA-seq, proteomics	Time course, 4 time points, 2 treatments,2–4 bio replicates	Mouse	Kidney	Individual	NO	Nearly	GEO, Proteome-Xchange	GEO	YES	Mouse Kidney FibrOmics browser, Search for expression patterns across omics

All papers carefully describe experimental designs, data acquisition and preprocessing pipelines, and share similar data management issues that are particularly relevant for this kind of studies, which we discuss below.

### Hosting of multi-omics data

The increasing complexity and size of multi-omics data has emerged as a major challenge with respect to hosting and accessing multi-omics analyses, as there is currently no unified public repository for multi-omics data. Consequently, none of the studies presented in this collection have deposited all their raw datasets into a single repository. The main reason for this is that most of the current omics data repositories were created in response to particular technologies available at the time, rather than with the vision of how such multi-dimensional data could be cohosted. Hence, public repositories have been designed according to data type (genomics, metabolomics, proteomics etc.) and assay type (array, sequencing, imaging etc.) and projects that generate all these multi-omics data have to deposit them accordingly. Moreover, well-defined repositories to host multivariate phenotypic data that may be collected in multi-omics projects do not exist. Although cross-referencing between repositories is possible and has already been implemented in many cases, this is still not available at the individual sample level, which is needed for many integrative analysis approaches. Establishing such links is not trivial. Many experimental designs are possible in multi-omics projects, as evidenced by this collection where we found matching across omics established for individual samples, for experimental conditions, and for multiple experimental batches or pooled samples. This current lack of public infrastructure has created an opportunity for commercial innovations such as cloud-based hosting and analysis platforms. Examples of such private initiatives include Lifebit (https://lifebit.ai/), Seven Bridges Genomics (https://www.sevenbridges.com/) and others who are already providing cloud-based platforms for hosting multi-omics data for integrative analysis. Software applications such as STATegraEMS^8^ have also been developed to address this same problem. To showcase the power of this approach, multi-omics data presented in the collection are being hosted on the Lifebit platform (https://opendata.lifebit.ai/) with free access. The field, however, is still in need of consistent standards and database protocols for hosting multi-omics data that can meaningfully address the complexity of all possible experimental designs.

### Completeness of multi-omics data

Multi-omics is essentially open-ended, so it is not surprising that most papers, excluding the PGP-UK pilot study of ten individuals, did not present a ‘complete’ multi-omics dataset for all samples included in the study. Sample availability, budget limitations or simply experimental constraints alone, frequently result in datasets with missing data for some omics. Similarly to the links across platforms, readily identifiable information of dataset completeness is important for reuse, as certain data analysis approaches will require complete or balanced designs. Although not included in this collection, imputation of missing data has improved significantly over the past years and may help to address this issue in the future^9,10^. Data hosting resources that provide adequate links across samples will help to identify the completeness of the multi-omics dataset. Alternatively, tools for data filtering as a function of the available information, in combination with phenotypic data, are extremely valuable for the reuse of these datasets. The Lifebit (https://lifebit.ai/) and ColPortal resources provide these functionalities.

### Quality control of multi-omics data

Quality control (QC) of the data is an essential requirement for a Data Descriptor and can be demonstrated by showing reproducibility of replicated measurements. In the case of multi-omics data, additional QC metrics should be considered that assess the relationship between datasets. These additional quality metrics are vital as omics technologies may vary in their accuracy, technical noise or signal dynamic range, and valid conclusions on integrative analysis can only be drawn when consistent quality is achieved across platforms. While all papers included in this collection include QC analyses, it is interesting to note how differently this was approached by the different studies, largely motivated by the type of project and goal of the study.

The PGP-UK paper focused on sample matching in large experiments to tackle the problem of potential mislabeling when processing many samples. Both the US Food and Drug Administration (FDA) and the National Cancer Institute (NCI) have recognized this problem and have recently launched a call to the scientific community to develop computational algorithms to detect and correct mislabeled samples in multi-omics datasets (https://precision.fda.gov/challenges/5). PGP-UK presents a strategy based on matching by genetic variability using single nucleotide polymorphisms (SNPs). Though matching by genotyping is possible for sequencing data, this strategy is only an option when the different omics are measured in the very same biological sample and there is genomic diversity among the samples – i.e. experiments do not use inbred organisims or cell lines. Moreover, this strategy would only work for sequencing, not for metabolomics or proteomics data.

Other approaches to demonstrate quality included showing agreement across omics in data variability patterns, such as by Principal Component Analysis, PCA (STATegra, ColPortal, Fibrotic Kidney). These results are interesting and valid when presented, but may not always be applicable, as there is no fundamental reason to believe that experimental factors will always affect different molecular levels in the same way. This was actually the case in the collection papers, where the PCA plots show similar, but not identical, grouping of samples by experimental condition.

Another type of validation presented by STATegra and Fibrotic Kidney was providing a proof that the dataset was able to recapitulate previous knowledge across multi-omics data for specific genes. Although, in a similar way, this might not be always possible in all multi-omics studies, it is very unlikely that a multi-omics study will be conducted on a system for which no previous knowledge exists and hence this type of validation data is broadly useful to check the consistency of the multi-omics dataset.

### Code

All papers include the code of their preprocessing or analysis pipelines as scripts. This adds important value to the collection, since the analysis code is not frequently included in genomics papers as this is not required by most journals in the field, hindering reproducibility of results. Providing analysis code as scripts, however, may not be as straight-forward as it seems, especially when different programming languages or platforms are combined in the analysis pipeline. For example, initial steps in proteomics or metabolomics data analysis typically use specialized or commercial software. Consequently, in these cases, only software parameters, but not the actual code can be reported. The collection addresses this software platform heterogeneity in different ways. STATegra provides full pipelines as consolidated text files where software parameters, command lines, and different languages are combined. This ensures full documentation. The code for each language, however, needs to be extracted from each script to be run. ColPortal and PVY only provide R scripts with statistical analysis code while previous steps are simply described in the methods section. An elaborated solution is presented in the Sleep Deprivation project, where the analysis pipeline differentiates code at three levels of preprocessing. The low-level layer is composed of scripts with heterogeneous languages tailored for each omics, while medium and high layer scripts include the statistical analysis performed in the same platforms. This facilitates reproducibility and re-running of analysis pipelines with different parameters or software versions.

### Resources for integrative data exploration

There are a large variety of tools for the integrative visualization of multi-omics datasets^11–16^, and large genomics projects have implemented solutions to visualize their multi-layered data. However, in this collection several papers include specific software developments that integrate data through molecular IDs (i.e., gene, protein or metabolite IDs) to facilitate browsing and visualization of the multi-layered information. This suggests that current public solutions that fundamentally focus on data deposition fall short in serving as portals for querying fully interconnected muti-omics data structures. This highlights once more the need of novel resources for improved accessibility and interoperability in the multi-omics data space.

### Conclusions and prospects

At a moment where multi-omics data structures are growing quickly and are being deployed for genomics medicine, this collection presents a unique compendium of datasets that can be used as a workbench for the development of software tools required by this type of data. Minimum information standards, currently available for single omics individually, are absent for multi-omics experiments. These should be created to capture the diversity in the relationship between samples, technologies, and data files that may be present in multi-omics projects. Additionally, novel hosting options that embrace the nature of the multi-platform and multi-layered data should become available. Note that this collection presents datasets that were obtained under a defined experimental setting and by one research team, hence data are comparable. Hosting multiple omics data types under the same umbrella will create opportunities to create multi-omics datasets by combining single-omics data from several studies. This will create new challenges for meta-data harmonization and control of batch effects that will need specific solutions. Finally, guidelines for quality and validation of data consistency need to be established to preserve the value of these datasets. This collection sets a precedent as to how to deal with these issues and hopefully will boost adoption of practices for better accessibility of multiomics datasets among the genomics community.
